# Supplementary material for: Heavy metal footprints in landfill-proximate soils of Jashore, Bangladesh: An index-based risk assessment
Source: PLoS One. 2026 May 21;21(5):e0349757. doi: 10.1371/journal.pone.0349757 (PMC13193546; doi:10.1371/journal.pone.0349757)
Supplement: S1 Table — (DOCX) [file pone.0349757.s001.docx]

**S1 Table. General characteristics of the sampling locations situated along the landfill.**

| **Sample ID** | **Land Use Type** | **Description** |
| --- | --- | --- |
| 1 | Agricultural Land | Site-1 is very close to the landfill and brinjal was cultivated there. It is on the north-east side of the plant. |
| 2 | Low agricultural Land | Site-2 is also very close to the landfill and arum was cultivated there. It is very on the north-east side of the plant. |
| 3 | Open field | Site-3 is located near the landfill. The sample was collected from a school field. |
| 4 | Woodland | Site-4 is a woodland near the landfill where mahogany is planted. |
| 5 | Low agricultural Land | Site-5 is on the southwest side and very close to the landfill. The soil is mixed with the ash of the waste material. |
| 6 | Paddy field | Site-6 is a paddy land |
| 7 | Agricultural land | Site-7 is situated very close to a brick field and it is near to the landfill and Industrial Area (IA). Corn was cultivated during sampling time. |
| 8 | Agricultural land | Site-8 is situated just near the River. Wastewater from the wastewater treatment plant and waste water from IA discharge into the river. Seasonal vegetables are cultivated in the land which is irrigated by river water. |
| 9 | Open field | Site-9 is located near the River. The sample was collected from the school field. |
| 10 | Industrial area | Site-10 is in the IA. |
| 11 | Open field | Site-11 is located far from the landfill. The sample was collected from the school field. |
| 12 | Paddy field | Site-12 is a paddy land. |
| 13 | Residential area | Site-13 is a Residential area far from the landfill. |
| 14 | Residential area | Site-14 is a Residential area close to the landfill. |
| 15 | Residential area | Site-15 is a Residential area close to the landfill. |
