# Supplementary material for: Heavy metal footprints in landfill-proximate soils of Jashore, Bangladesh: An index-based risk assessment
Source: PLoS One. 2026 May 21;21(5):e0349757. doi: 10.1371/journal.pone.0349757 (PMC13193546; doi:10.1371/journal.pone.0349757)
Supplement: S2 Table — B. Operating Conditions of NexION ICP-MS Instrument and Atomic Absorption Spectrophotometer. (DOCX) [file pone.0349757.s002.docx]

**S2A** **Table.** Limits of Detection (LOD) and quantification (LOQ) for elements.

| **Element** | **Unit** | **LOD** | **LOQ** |
| --- | --- | --- | --- |
| As | (µg/L) | 0.0121 | 0.121 |
| Hg | (µg/L) | 0.0013 | 0.012 |
| Cd | (µg/L) | 0.0052 | 0.053 |
| Pb | (µg/L) | 0.029 | 0.29 |
| Cr | (µg/L) | 0.063 | 0.62 |
| Zn | (µg/L) | 0.11 | 0.98 |
| Co | (µg/L) | 0.012 | 0.13 |
| Ni | (µg/L) | 0.067 | 0.67 |
| Cu | (µg/L) | 0.051 | 0.49 |
| Mn | (µg/L) | 0.072 | 0.73 |
| Fe | (µg/L) | 0.12 | 1.02 |

**S2B Table. Operating Conditions of NexION ICP-MS Instrument and Atomic Absorption Spectrophotometer.**

| **Inductively Coupled Plasma Mass Spectrometer (ICP-MS, NexION 2000, Perkin Elmer, USA)** | | **Atomic Absorption Spectrophotometer (Shimadzu AA-7000, Japan)** | |
| --- | --- | --- | --- |
| Operating Conditions | Type or Value | Operating Conditions | Type or Value |
| RF Power | 1600 W | Acetylene | 2.0 L/min |
| Plasma Gas Flow | 15 L/min | Air | 15 L/min |
| Auxiliary Gas Flow | 1.2 L/min | Wave  length | 213.9 nm |
| Nebulizer Gas Flow | Optimized for CeO^+^/Ce^+^ < 2.5%; Ce^++^/Ce < 2.5% | Lamp  Current | 08 mA |
| Cell Gas | Oxygen, helium | Slith  width | 0.7 nm |
|  |  | Detection  limit | 0.002 mg/L |
