# Supplementary material for: Heavy metal footprints in landfill-proximate soils of Jashore, Bangladesh: An index-based risk assessment
Source: PLoS One. 2026 May 21;21(5):e0349757. doi: 10.1371/journal.pone.0349757 (PMC13193546; doi:10.1371/journal.pone.0349757)
Supplement: S3 Table — (DOCX) [file pone.0349757.s003.docx]

**S3 Table. Classification of indices for the assessment of soil pollution.**

| **Contamination factor (CF)** | | |
| --- | --- | --- |
| **Category** | **Degree of contamination** | **References** |
| CF < 1 | Low contamination | [1,2] |
| 1 ≤ CF < 3 | Moderate contamination |  |
| 3 ≤ CF < 6 | Considerable contamination |  |
| CF > 6 | Very high contamination |  |
| **Enrichment factor (EF)** | | |
| **Category** | **Degree of enrichment** | **Reference** |
| <2 | Deficiency to minimal enrichment | [3] |
| 2-5 | Moderate enrichment |  |
| 5-10 | Severe enrichment |  |
| >10 | Very severe enrichment |  |
| **Geo-accumulation Index** | | |
| **Category** | **Degree of pollution** | **Reference** |
| I_geo_ ≤ 0 | Practically unpolluted | [4] |
| 0 < I_geo_ ≤ 1 | Slight to moderately polluted |  |
| 1 < I_geo_ ≤ 2 | Moderately polluted |  |
| 2 < I_geo_ ≤ 3 | Moderately to heavily polluted |  |
| 3 < I_geo_ ≤ 4 | Heavily polluted |  |
| 4 < I_geo_ ≤ 5 | Heavily to extremely polluted |  |
| 5 < I_geo_ | Extremely polluted |  |
|  | **Ecological risk factor (Er)** | **References** |
| **Category** | **Degree of contamination** | [5,6] |
| Er < 40 | Low potential ecological risk |  |
| 40 ≤ Er < 80 | Moderate potential ecological risk |  |
| 80 ≤ Er< 160 | Considerable potential ecological risk |  |
| 160 ≤ Er < 320 | High potential ecological risk |  |
| Er ≥ 320 | Very high ecological risk |  |
|  | **Potential ecological risk index (RI)** |  |
| **Category** | **Degree of contamination** | **References** |
| RI < 50 | Low ecological risk potential | [5,7] |
| 50 ≤ RI < 100 | Moderate potential ecological risk |  |
| 100 ≤ RI < 200 | Considerable high potential ecological risk |  |
| RI ≥ 200 | Significantly high potential ecological risk |  |

**References**

1. Sadhu K, Adhikari K, Gangopadhyay A. Assessment of Heavy Metal Contamination of Soils In and Around Open Cast Mines of Raniganj Area, India. 2012;1.

2. Likuku AS, Mmolawa KB, Gaboutloeloe GK. Assessment of Heavy Metal Enrichment and Degree of Contamination around the Copper-Nickel Mine in the Selebi Phikwe Region, Eastern Botswana. Environment and Ecology Research. 2013;1: 32–40. doi:10.13189/eer.2013.010202

3. Birch GF, Olmos MA. Sediment-bound heavy metals as indicators of human influence and biological risk in coastal water bodies. ICES Journal of Marine Science. 2008;65: 1407–1413. doi:10.1093/icesjms/fsn139

4. Ma X, Zuo H, Tian M, Zhang L, Meng J, Zhou X, et al. Assessment of heavy metals contamination in sediments from three adjacent regions of the Yellow River using metal chemical fractions and multivariate analysis techniques. Chemosphere. 2016;144: 264–272. doi:10.1016/j.chemosphere.2015.08.026

5. Hakanson L. An ecological risk index for aquatic pollution control.a sedimentological approach. Water Research. 1980;14: 975–1001. doi:10.1016/0043-1354(80)90143-8

6. Wu Q, Leung JYS, Geng X, Chen S, Huang X, Li H, et al. Heavy metal contamination of soil and water in the vicinity of an abandoned e-waste recycling site: implications for dissemination of heavy metals. Sci Total Environ. 2015;506–507: 217–225. doi:10.1016/j.scitotenv.2014.10.121

7. Gbadamosi MR, Afolabi TA, Ogunneye AL, Ogunbanjo OO, Omotola EO, Kadiri TM, et al. Distribution of radionuclides and heavy metals in the bituminous sand deposit in Ogun State, Nigeria – A multi-dimensional pollution, health and radiological risk assessment. Journal of Geochemical Exploration. 2018;190: 187–199. doi:10.1016/j.gexplo.2018.03.006
