# Supplementary material for: Heavy metal footprints in landfill-proximate soils of Jashore, Bangladesh: An index-based risk assessment
Source: PLoS One. 2026 May 21;21(5):e0349757. doi: 10.1371/journal.pone.0349757 (PMC13193546; doi:10.1371/journal.pone.0349757)
Supplement: S4 Table — (DOCX) [file pone.0349757.s004.docx]

**S4 Table. Parameters used to evaluate the human health risk of heavy metals in soil samples.**

| **Parameters** | **Unit** | **Child** | **Adult** | **Reference** |
| --- | --- | --- | --- | --- |
| Non-radioactive metal concentration in soil (Cs) | mg/kg | Observed Concentration | Observed Concentration | This study |
| Soil ingestion rate (IngR) | mg day^-1^ | 200 | 100 | [1–4] |
| Soil inhalation rate (InhR) | m^3^ day^-1^ | 10 | 20 |  |
| Exposure frequency (EF) | day/year | 350 | 350 |  |
| Exposure duration (ED) | year | 6 | 30 |  |
| Body weight (BW) | Kg | 15 | 70 |  |
| Particle emission factor (PEF) | m^3^ kg^-1^ | 1.3 *×* 10^9^ | 1.3 *×* 10^9^ |  |
| Skin area (SA) | cm^2^ | 2800 | 5800 |  |
| Skin adherence factor (AF) | mg/cm^2^ | 0.2 | 0.07 |  |
| Conversion factor (CF) | none | 1.0 × 10^−6^ | 1.0 × 10^−6^ |  |
| Dermal absorption factor (ABS) | none | 0.001 | 0.001 |  |
| Averaging time for non- carcinogen (AT) | day | ED × 365 | ED × 365 |  |
| Averaging time for carcinogen (AT) | day | 70 × 365 | 70 × 365 |  |
| Chronic reference dose (Rfd) | mg kg^-1^ day^-1^ | Ingestion Rfd: 4.0 × 10^−3^ (Pb), 1.00 × 10^−3^ (Cd), 3.00 ×10^−3^ (Cr), 3.00 × 10^−4^ (As), 4.60× 10^−2^ (Mn), 0.7 (Fe), 2.00 × 10^−2^ (Co), 2.00 × 10^−2^ (Ni), 4.00 × 10^−2^ (Cu), 3.00 × 10^−1^ (Zn). | | [2,5,6] |
|  |  | Inhalation Rfd: 3.25 × 10^−3^ (Pb), 1× 10^−3^ (Cd), 2.86 ×10^−5^ (Cr), 3.01 × 10^−4^ (As), 1.43× 10^−5^ (Mn), 5.71 × 10^−6^ (Co), 2.06 × 10^−2^ (Ni), 4.02 × 10^−2^ (Cu), 3.00 × 10^−1^ (Zn), 3× 10^−1^ (Fe). | |  |
|  |  | Dermal Rfd: 5.25 × 10^−4^ (Pb), 1.0 × 10^−5^ (Cd), 6.0 ×10^−5^ (Cr), 1.23 × 10^−4^ (As), 1.84× 10^−3^ (Mn), 0.14 (Fe), 5.70 × 10^−6^ (Co), 5.40 × 10^−5^ (Ni), 1.2 × 10^−2^ (Cu), 6.00 × 10^−6^ (Zn). | |  |
| Carcinogenic slope factor  SF | (mg kg^-1^ day^-1^)^-1^ | Ingestion SF: 1.50 (As), 5.01× 10^−1^ (Cr), 0.38 (Cd), 1.7 (Ni), 8.5 × 10^−3^ (Pb) | | [5,7] |
|  |  | Inhalation SF: 1.51 × 10^1^ (As), 6.30 (Cd), 0.42 (Cr), 0.84 (Ni), 4.2 × 10^−2^ (Pb) | |  |
|  |  | Dermal SF: 3.66 (As), 2.0 (Cr), 6.3 (Cd), 8.40×10^1^ (Ni), 8.5 × 10^−6^ (Pb) | |  |

**References**

1. Karimi A, Naghizadeh A, Biglari H, Peirovi R, Ghasemi A, Zarei A. Assessment of human health risks and pollution index for heavy metals in farmlands irrigated by effluents of stabilization ponds. Environ Sci Pollut Res Int. 2020;27: 10317–10327. doi:10.1007/s11356-020-07642-6

2. De Miguel E, Iribarren I, Chacón E, Ordoñez A, Charlesworth S. Risk-based evaluation of the exposure of children to trace elements in playgrounds in Madrid (Spain). Chemosphere. 2007;66: 505–513. doi:10.1016/j.chemosphere.2006.05.065

3. Wang J, Liu G, Liu H, Lam PKS. Multivariate statistical evaluation of dissolved trace elements and a water quality assessment in the middle reaches of Huaihe River, Anhui, China. Sci Total Environ. 2017;583: 421–431. doi:10.1016/j.scitotenv.2017.01.088

4. Bai H, Hu B, Wang C, Bao S, Sai G, Xu X, et al. Assessment of Radioactive Materials and Heavy Metals in the Surface Soil around the Bayanwula Prospective Uranium Mining Area in China. IJERPH. 2017;14: 300. doi:10.3390/ijerph14030300

5. Gad A, Saleh A, Farhat HI, Dawood YH, Abd El Bakey SM. Spatial Distribution, Contamination Levels, and Health Risk Assessment of Potentially Toxic Elements in Household Dust in Cairo City, Egypt. Toxics. 2022;10: 466. doi:10.3390/toxics10080466

6. Ferreira-Baptista L, De Miguel E. Geochemistry and risk assessment of street dust in Luanda, Angola: A tropical urban environment. Atmospheric Environment. 2005;39: 4501–4512. doi:10.1016/j.atmosenv.2005.03.026

7. Mugudamani I, Oke SA, Gumede TP. Influence of Urban Informal Settlements on Trace Element Accumulation in Road Dust and Their Possible Health Implications in Ekurhuleni Metropolitan Municipality, South Africa. Toxics. 2022;10: 253. doi:10.3390/toxics10050253
