# Supplementary material for: Heavy metal footprints in landfill-proximate soils of Jashore, Bangladesh: An index-based risk assessment
Source: PLoS One. 2026 May 21;21(5):e0349757. doi: 10.1371/journal.pone.0349757 (PMC13193546; doi:10.1371/journal.pone.0349757)
Supplement: S5 Table — (DOCX) [file pone.0349757.s005.docx]

**S5 Table. Metal contamination factors (CFs) and pollution load indices (PLIs) for the soils at all the sites tested along the landfill.**

| ID name | Contamination factors (CFs) | | | | | | | | | | | PLI |
| --- | --- | --- | --- | --- | --- | --- | --- | --- | --- | --- | --- | --- |
|  | As | Hg | Cd | Pb | Cr | Zn | Co | Ni | Cu | Mn | Fe |  |
| 1 | 1.00 | 3.34 | 3.79 | 1.58 | 0.48 | 2.14 | 0.81 | 0.77 | 6.24 | 0.70 | 0.54 | 1.36 |
| 2 | 0.75 | 3.04 | 1.76 | 1.44 | 0.45 | 1.78 | 0.81 | 0.74 | 5.40 | 0.66 | 0.53 | 1.16 |
| 3 | 0.60 | 2.13 | 1.33 | 1.08 | 0.42 | 2.15 | 0.73 | 0.62 | 4.21 | 0.49 | 0.45 | 0.97 |
| 4 | 0.94 | 0.61 | 0.93 | 1.32 | 0.45 | 1.23 | 0.84 | 0.79 | 5.08 | 0.83 | 0.49 | 0.94 |
| 5 | 1.16 | 3.77 | 5.89 | 3.60 | 0.73 | 7.18 | 0.74 | 0.96 | 7.96 | 0.82 | 0.54 | 1.91 |
| 6 | 1.02 | 0.61 | 0.93 | 2.72 | 0.48 | 1.63 | 0.90 | 0.76 | 5.17 | 0.57 | 0.59 | 1.04 |
| 7 | 0.91 | 0.29 | 0.68 | 0.99 | 0.42 | 1.51 | 0.71 | 0.60 | 3.87 | 0.57 | 0.47 | 0.76 |
| 8 | 1.91 | 1.52 | 0.91 | 1.57 | 0.69 | 1.40 | 0.90 | 0.97 | 6.15 | 1.25 | 0.65 | 1.30 |
| 9 | 0.92 | 1.51 | 4.23 | 2.23 | 0.50 | 1.78 | 0.93 | 1.49 | 5.06 | 0.86 | 0.52 | 1.39 |
| 10 | 0.54 | 0.61 | 3.82 | 2.56 | 0.49 | 4.48 | 0.85 | 0.74 | 7.73 | 0.43 | 0.48 | 1.20 |
| 11 | 0.99 | 0.29 | 1.01 | 1.36 | 0.54 | 1.59 | 1.02 | 0.90 | 6.04 | 0.98 | 0.65 | 1.02 |
| 12 | 1.05 | 1.52 | 2.24 | 1.71 | 0.71 | 1.67 | 1.15 | 1.05 | 7.61 | 0.53 | 0.77 | 1.35 |
| 13 | 0.89 | 0.91 | 1.04 | 1.73 | 0.57 | 2.43 | 1.02 | 0.83 | 6.04 | 0.91 | 0.62 | 1.18 |
| 14 | 0.52 | 1.21 | 0.76 | 1.78 | 0.37 | 2.12 | 0.58 | 0.54 | 4.55 | 0.51 | 0.50 | 0.88 |
| 15 | 0.61 | 1.52 | 0.94 | 2.38 | 0.45 | 8.01 | 0.58 | 0.51 | 5.73 | 0.62 | 0.42 | 1.12 |
| Mean | 0.92 | 1.52 | 2.02 | 1.87 | 0.521 | 2.74 | 0.84 | 0.82 | 5.79 | 0.72 | 0.55 | 1.17 |
| Min | 0.52 | 0.29 | 0.76 | 1.08 | 0.37 | 1.23 | 0.58 | 0.51 | 3.87 | 0.43 | 0.42 | 0.76 |
| Max | 1.91 | 3.34 | 4.23 | 3.60 | 0.73 | 8.01 | 1.15 | 1.49 | 7.96 | 1.25 | 0.77 | 1.91 |
