# Supplementary material for: Heavy metal footprints in landfill-proximate soils of Jashore, Bangladesh: An index-based risk assessment
Source: PLoS One. 2026 May 21;21(5):e0349757. doi: 10.1371/journal.pone.0349757 (PMC13193546; doi:10.1371/journal.pone.0349757)
Supplement: S6 Table — (DOCX) [file pone.0349757.s006.docx]

**S6 Table. Results of enrichment factors (EF) and geo-accumulation indices (I_geo_) of heavy metals in soils of the landfill area, Bangladesh.**

| **ID** | **As** | | **Hg** | | **Cd** | | **Pb** | | **Cr** | | **Zn** | | **Co** | | **Ni** | | **Cu** | | **Mn** | | **Fe** |
| --- | --- | --- | --- | --- | --- | --- | --- | --- | --- | --- | --- | --- | --- | --- | --- | --- | --- | --- | --- | --- | --- |
|  | **EF** | **I_geo_** | **EF** | **I_geo_** | **EF** | **I_geo_** | **EF** | **I_geo_** | **EF** | **I_geo_** | **EF** | **I_geo_** | **EF** | **I_geo_** | **EF** | **I_geo_** | **EF** | **I_geo_** | **EF** | **I_geo_** | **I_geo_** |
| 1 | 1.82 | -0.58 | 6.10 | 1.16 | 6.92 | 1.34 | 2.89 | 0.08 | 0.88 | -1.63 | 3.92 | 0.52 | 1.50 | -0.87 | 1.41 | -0.95 | 11.4 | 2.06 | 1.29 | -1.09 | -1.45 |
| 2 | 1.42 | -0.98 | 5.71 | 1.02 | 3.31 | 0.23 | 2.71 | -0.05 | 0.85 | -1.72 | 3.34 | 0.25 | 1.54 | -0.87 | 1.40 | -1.01 | 10.1 | 1.85 | 1.25 | -1.17 | -1.49 |
| 3 | 1.32 | -1.32 | 4.71 | 0.51 | 2.94 | -0.17 | 2.39 | -0.47 | 0.93 | -1.84 | 4.74 | 0.52 | 1.62 | -1.03 | 1.37 | -1.27 | 9.30 | 1.49 | 1.10 | -1.59 | -1.73 |
| 4 | 1.89 | -0.66 | 1.23 | -1.29 | 1.87 | -0.69 | 2.66 | -0.18 | 0.91 | -1.73 | 2.48 | -0.28 | 1.69 | -0.84 | 1.60 | -0.91 | 10.2 | 1.76 | 1.68 | -0.84 | -1.59 |
| 5 | 2.14 | -0.36 | 6.93 | 1.33 | 10.8 | 1.97 | 6.62 | 1.27 | 1.35 | -1.03 | 13.1 | 2.26 | 1.37 | -1.01 | 1.78 | -0.63 | 14.6 | 2.41 | 1.51 | -0.86 | -1.46 |
| 6 | 1.72 | -0.55 | 1.03 | -1.29 | 1.58 | -0.68 | 4.61 | 0.86 | 0.83 | -1.62 | 2.77 | 0.13 | 1.53 | -0.73 | 1.30 | -0.97 | 8.75 | 1.79 | 0.98 | -1.37 | -1.34 |
| 7 | 1.93 | -0.72 | 0.64 | -2.32 | 1.45 | -1.14 | 2.13 | -0.59 | 0.91 | -1.81 | 3.22 | 0.01 | 1.51 | -1.08 | 1.29 | -1.30 | 8.25 | 1.37 | 1.23 | -1.38 | -1.67 |
| 8 | 2.92 | 0.35 | 2.32 | 0.02 | 1.40 | -0.71 | 2.40 | 0.07 | 1.07 | -1.10 | 2.15 | -0.09 | 1.39 | -0.72 | 1.49 | -0.62 | 9.39 | 2.04 | 1.92 | -0.26 | -1.19 |
| 9 | 1.75 | -0.69 | 2.86 | 1.12 | 7.99 | 1.50 | 4.21 | 0.57 | 0.95 | -1.58 | 3.38 | 0.25 | 1.77 | -0.68 | 2.81 | -0.01 | 9.56 | 1.76 | 1.63 | -0.79 | -1.50 |
| 10 | 1.11 | -1.47 | 1.25 | -1.29 | 7.84 | 1.35 | 5.26 | 0.78 | 1.01 | -1.61 | 9.18 | 1.58 | 1.75 | -0.81 | 1.53 | -1.00 | 15.8 | 2.37 | 0.89 | -1.78 | -1.62 |
| 11 | 1.51 | -0.60 | 0.46 | -2.32 | 1.55 | -0.56 | 2.09 | -0.13 | 0.83 | -1.47 | 2.44 | 0.09 | 1.57 | -0.55 | 1.39 | -0.72 | 9.23 | 2.01 | 1.50 | -0.61 | -1.20 |
| 12 | 1.36 | -0.51 | 1.97 | 0.02 | 2.91 | 0.58 | 2.21 | 0.19 | 0.92 | -1.08 | 2.17 | 0.16 | 1.50 | -0.37 | 1.36 | -0.51 | 9.86 | 2.34 | 0.70 | -1.48 | -0.96 |
| 13 | 1.42 | -0.75 | 1.45 | -0.72 | 1.67 | -0.52 | 2.77 | 0.21 | 0.92 | -1.38 | 3.87 | 0.70 | 1.64 | -0.54 | 1.33 | -0.84 | 9.63 | 2.01 | 1.45 | -0.72 | -1.26 |
| 14 | 1.05 | -1.50 | 2.42 | -0.31 | 1.53 | -0.97 | 3.57 | 0.25 | 0.75 | -2.01 | 4.25 | 0.50 | 1.18 | -1.35 | 1.09 | -1.46 | 9.11 | 1.60 | 1.03 | -1.54 | -1.58 |
| 15 | 1.45 | -1.29 | 3.62 | 0.02 | 2.25 | -0.66 | 5.67 | 0.67 | 1.09 | -1.71 | 19.0 | 2.42 | 1.39 | -1.36 | 1.22 | -1.54 | 13.6 | 1.93 | 1.48 | -1.26 | -1.83 |
