# Supplementary material for: Heavy metal footprints in landfill-proximate soils of Jashore, Bangladesh: An index-based risk assessment
Source: PLoS One. 2026 May 21;21(5):e0349757. doi: 10.1371/journal.pone.0349757 (PMC13193546; doi:10.1371/journal.pone.0349757)
Supplement: S7 Table — (DOCX) [file pone.0349757.s007.docx]

**S7 Table. Results of toxic unit (TU) of heavy metals in soils of the landfill area, Bangladesh.**

| **ID Name** | **Toxic Unit (TU)** | | | | | | | |
| --- | --- | --- | --- | --- | --- | --- | --- | --- |
|  | **As** | **Hg** | **Cd** | **Pb** | **Cr** | **Ni** | **Cu** | **∑TU** |
| 1 | 0.77 | 2.75 | 0.32 | 0.35 | 0.48 | 1.03 | 1.43 | 7.13 |
| 2 | 0.58 | 2.51 | 0.15 | 0.32 | 0.46 | 1.00 | 1.23 | 6.24 |
| 3 | 0.46 | 1.76 | 0.11 | 0.24 | 0.42 | 0.83 | 0.96 | 4.78 |
| 4 | 0.72 | 0.50 | 0.08 | 0.29 | 0.45 | 1.06 | 1.16 | 4.27 |
| 5 | 0.89 | 3.11 | 0.50 | 0.79 | 0.74 | 1.29 | 1.82 | 9.14 |
| 6 | 0.78 | 0.50 | 0.08 | 0.60 | 0.49 | 1.02 | 1.18 | 4.66 |
| 7 | 0.70 | 0.25 | 0.06 | 0.22 | 0.43 | 0.81 | 0.89 | 3.34 |
| 8 | 1.46 | 1.25 | 0.08 | 0.34 | 0.70 | 1.30 | 1.41 | 6.55 |
| 9 | 0.71 | 1.25 | 0.36 | 0.49 | 0.50 | 1.99 | 1.16 | 6.46 |
| 10 | 0.41 | 0.50 | 0.33 | 0.56 | 0.49 | 1.00 | 1.77 | 5.06 |
| 11 | 0.76 | 0.25 | 0.09 | 0.30 | 0.54 | 1.21 | 1.38 | 4.52 |
| 12 | 0.81 | 1.25 | 0.19 | 0.38 | 0.71 | 1.41 | 1.74 | 6.48 |
| 13 | 0.68 | 0.75 | 0.09 | 0.38 | 0.58 | 1.12 | 1.38 | 4.98 |
| 14 | 0.41 | 1.00 | 0.07 | 0.39 | 0.37 | 0.73 | 1.04 | 4.00 |
| 15 | 0.47 | 1.25 | 0.08 | 0.52 | 0.46 | 0.69 | 1.31 | 4.78 |
| Mean | 0.707 | 1.26 | 0.178 | 0.410 | 0.521 | 1.09 | 1.32 | 5.50 |
