# Supplementary material for: Heavy metal footprints in landfill-proximate soils of Jashore, Bangladesh: An index-based risk assessment
Source: PLoS One. 2026 May 21;21(5):e0349757. doi: 10.1371/journal.pone.0349757 (PMC13193546; doi:10.1371/journal.pone.0349757)
Supplement: S8 Table — (DOCX) [file pone.0349757.s008.docx]

**S8 Table. Ecological risk factors (Er) and potential ecological risk indices (RI) of heavy metals in soils of the landfill area, Bangladesh.**

| **ID Name** | **Ecological Risk Factor (Er)** | | | | | | | | | | | |
| --- | --- | --- | --- | --- | --- | --- | --- | --- | --- | --- | --- | --- |
|  | **As** | **Hg** | **Cd** | **Pb** | **Cr** | **Zn** | **Co** | **Ni** | **Cu** | **Mn** | **Fe** | **RI** |
| 1 | 10.01 | 133.68 | 113.73 | 7.92 | 0.97 | 2.15 | 4.10 | 3.87 | 31.21 | 0.70 | 1.10 | 309.44 |
| 2 | 7.59 | 121.77 | 52.90 | 7.22 | 0.91 | 1.78 | 4.10 | 3.74 | 27.02 | 0.67 | 1.07 | 228.75 |
| 3 | 6.02 | 85.45 | 39.98 | 5.43 | 0.84 | 2.15 | 3.66 | 3.12 | 21.09 | 0.50 | 0.91 | 169.14 |
| 4 | 9.46 | 24.48 | 27.98 | 6.63 | 0.90 | 1.24 | 4.20 | 3.98 | 25.42 | 0.84 | 1.00 | 106.13 |
| 5 | 11.67 | 151.00 | 176.78 | 18.03 | 1.47 | 7.18 | 3.73 | 4.84 | 39.83 | 0.82 | 1.09 | 416.45 |
| 6 | 10.22 | 24.49 | 27.99 | 13.64 | 0.98 | 1.64 | 4.52 | 3.84 | 25.90 | 0.58 | 1.18 | 114.98 |
| 7 | 9.11 | 11.98 | 20.46 | 5.00 | 0.85 | 1.52 | 3.55 | 3.04 | 19.39 | 0.58 | 0.94 | 76.42 |
| 8 | 19.14 | 60.91 | 27.46 | 7.86 | 1.40 | 1.41 | 4.54 | 4.88 | 30.78 | 1.26 | 1.31 | 160.95 |
| 9 | 9.29 | 60.72 | 127.00 | 11.15 | 1.00 | 2.00 | 4.68 | 7.45 | 25.33 | 1.05 | 1.06 | 250.74 |
| 10 | 5.43 | 24.46 | 114.82 | 12.83 | 0.98 | 4.48 | 4.27 | 3.74 | 38.68 | 0.44 | 0.98 | 211.12 |
| 11 | 9.92 | 11.99 | 30.46 | 6.85 | 1.08 | 1.60 | 5.14 | 4.54 | 30.21 | 0.98 | 1.31 | 104.09 |
| 12 | 10.53 | 60.89 | 67.38 | 8.56 | 1.42 | 1.68 | 5.79 | 5.27 | 38.10 | 0.54 | 1.55 | 201.70 |
| 13 | 8.94 | 36.42 | 31.43 | 8.70 | 1.15 | 2.43 | 5.14 | 4.18 | 30.24 | 0.91 | 1.26 | 130.80 |
| 14 | 5.30 | 48.40 | 22.95 | 8.94 | 0.75 | 2.13 | 2.94 | 2.72 | 22.80 | 0.52 | 1.00 | 118.45 |
| 15 | 6.13 | 60.95 | 28.47 | 11.94 | 0.92 | 8.01 | 2.93 | 2.58 | 28.66 | 0.62 | 0.84 | 152.04 |
| Mean | 9.25 | 61.17 | 60.65 | 9.38 | 1.04 | 2.76 | 4.22 | 4.12 | 28.98 | 0.73 | 1.11 | 183.41 |
