# Supplementary material for: Heavy metal footprints in landfill-proximate soils of Jashore, Bangladesh: An index-based risk assessment
Source: PLoS One. 2026 May 21;21(5):e0349757. doi: 10.1371/journal.pone.0349757 (PMC13193546; doi:10.1371/journal.pone.0349757)
Supplement: S9 Table — B. Assessment of the carcinogenic risks to human health caused by ingestion, inhalation, and dermal exposure in children and adults residing in the vicinity of the landfill in Bangladesh. (DOCX) [file pone.0349757.s009.docx]

**S9A Table. Assessment of non-carcinogenic human health risks for children and adults in the vicinity of the landfill, Jashore in Bangladesh, considering the potential exposure by ingestion, inhalation, and Dermal pathways.**

| **Non-Carcinogenic Risk** | | **Children** | | | | **Adults** | | | |
| --- | --- | --- | --- | --- | --- | --- | --- | --- | --- |
|  |  | **HQ _ingestion_** | **HQ _Inhalation_** | **HQ _Dermal_** | **HI= ∑HQ** | **HQ _ingestion_** | **HQ _Inhalation_** | **HQ _Dermal_** | **HI =∑HQ** |
| Pb | Min | 6.39E-02 | 3.02E-12 | 1.36E-03 | 6.53E-02 | 2.93E-03 | 5.56E-13 | 2.12E-04 | 3.15E-03 |
|  | Max | 2.30E-01 | 1.09E-11 | 4.92E-03 | 2.35E-01 | 1.06E-02 | 2E-12 | 7.64E-04 | 1.13E-02 |
|  | Mean | 1.20E-01 | 5.68E-12 | 2.56E-03 | 1.22E-01 | 5.51E-03 | 1.04E-12 | 3.97E-04 | 5.90E-03 |
| Cd | Min | 2.62E-03 | 1.01E-13 | 7.32E-04 | 3.35E-03 | 1.20E-04 | 1.85E-14 | 1.14E-04 | 2.34E-04 |
|  | Max | 2.26E-02 | 8.69E-13 | 6.33E-03 | 2.89E-02 | 1.04E-03 | 1.6E-13 | 9.83E-04 | 2.02E-03 |
|  | Mean | 7.75E-03 | 2.98E-13 | 2.17E-03 | 9.93E-03 | 3.56E-04 | 5.48E-14 | 3.37E-04 | 6.93E-04 |
| Cr | Min | 1.43E-01 | 1.43E-01 | 5.78E-10 | 1.63E-01 | 6.58E-03 | 1.06E-10 | 3.12E-03 | 9.70E-03 |
|  | Max | 2.82E-01 | 2.82E-01 | 1.14E-09 | 3.22E-01 | 1.30E-02 | 2.09E-10 | 6.14E-03 | 1.91E-02 |
|  | Mean | 2.00E-01 | 2.00E-01 | 8.06E-10 | 2.28E-01 | 9.18E-03 | 1.48E-10 | 4.35E-03 | 1.35E-02 |
| Hg | Min | 5.10E-03 | 6.85E-13 | 1.43E-05 | 5.12E-03 | 2.34E-04 | 1.26E-13 | 2.22E-06 | 2.37E-04 |
|  | Max | 6.44E-02 | 8.63E-12 | 1.80E-04 | 6.45E-02 | 2.95E-03 | 1.59E-12 | 2.8E-05 | 2.98E-03 |
|  | Mean | 2.81E-02 | 3.76E-12 | 7.86E-05 | 2.81E-02 | 1.29E-03 | 6.91E-13 | 1.22E-05 | 1.30E-03 |
| As | Min | 2.93E-01 | 1.13E-11 | 2.00E-03 | 2.95E-01 | 1.35E-02 | 2.07E-12 | 3.11E-04 | 1.38E-02 |
|  | Max | 1.06E+00 | 4.07E-11 | 7.24E-03 | 1.07E+00 | 4.87E-02 | 7.47E-12 | 1.13E-03 | 4.98E-02 |
|  | Mean | 5.13E-01 | 1.96E-11 | 3.50E-03 | 5.16E-01 | 2.35E-02 | 3.61E-12 | 5.44E-04 | 2.41E-02 |
| Fe | Min | 3.63E-01 | 3.26E-11 | 5.08E-03 | 3.68E-01 | 1.67E-02 | 5.99E-12 | 7.90E-04 | 1.75E-02 |
|  | Max | 6.66E-01 | 5.98E-11 | 9.33E-03 | 6.76E-01 | 3.06E-02 | 1.1E-11 | 1.45E-03 | 3.20E-02 |
|  | Mean | 4.76E-01 | 4.28E-11 | 6.67E-03 | 4.83E-01 | 2.19E-02 | 7.85E-12 | 1.04E-03 | 2.29E-02 |
| Mn | Min | 1.03E-01 | 1.27E-08 | 7.21E-03 | 1.10E-01 | 4.73E-03 | 2.34E-09 | 1.12E-03 | 5.85E-03 |
|  | Max | 2.97E-01 | 3.67E-08 | 2.08E-02 | 3.18E-01 | 1.36E-02 | 6.75E-09 | 3.23E-03 | 1.69E-02 |
|  | Mean | 1.70E-01 | 2.11E-08 | 1.19E-02 | 1.82E-01 | 7.83E-03 | 3.87E-09 | 1.85E-03 | 9.68E-03 |
| Zn | Min | 5.01E-03 | 1.93E-13 | 7.01E-01 | 7.06E-01 | 2.30E-04 | 3.54E-14 | 1.09E-01 | 1.09E-01 |
|  | Max | 3.24E-02 | 1.25E-12 | 4.54E+00 | 4.57E+00 | 1.49E-03 | 2.29E-13 | 7.05E-01 | 7.07E-01 |
|  | Mean | 1.11E-02 | 4.27E-13 | 1.56E+00 | 1.57E+00 | 5.10E-04 | 7.85E-14 | 2.42E-01 | 2.42E-01 |
| Co | Min | 7.12E-03 | 9.59E-10 | 6.99E-02 | 7.70E-02 | 3.27E-04 | 1.76E-10 | 1.76E-10 | 1.12E-02 |
|  | Max | 1.41E-02 | 1.89E-09 | 1.38E-01 | 1.52E-01 | 6.46E-04 | 3.48E-10 | 3.48E-10 | 2.21E-02 |
|  | Mean | 1.03E-02 | 1.38E-09 | 1.01E-01 | 1.11E-01 | 4.71E-04 | 2.54E-10 | 2.54E-10 | 1.60E-02 |
| Ni | Min | 1.58E-02 | 6.96E-13 | 1.93E-02 | 3.80E-02 | 7.26E-04 | 1.08E-13 | 2.55E-03 | 3.27E-03 |
|  | Max | 4.57E-02 | 1.71E-12 | 4.74E-02 | 9.32E-02 | 2.10E-03 | 3.14E-13 | 7.37E-03 | 9.47E-03 |
|  | Mean | 2.53E-02 | 9.44E-13 | 2.62E-02 | 5.15E-02 | 1.16E-03 | 1.73E-13 | 4.07E-03 | 5.23E-03 |
| Cu | Min | 5.58E-02 | 2.14E-12 | 5.21E-04 | 5.63E-02 | 2.56E-03 | 3.92E-13 | 8.09E-05 | 2.64E-03 |
|  | Max | 1.15E-01 | 4.39E-12 | 1.07E-03 | 1.16E-01 | 5.26E-03 | 8.05E-13 | 1.66E-04 | 5.43E-03 |
|  | Mean | 8.34E-02 | 3.19E-12 | 7.78E-04 | 8.41E-02 | 3.83E-03 | 5.86E-13 | 1.21E-04 | 3.95E-03 |

**S9B Table. Assessment of the carcinogenic risks to human health caused by ingestion, inhalation, and dermal exposure in children and adults residing in the vicinity of the landfill in Bangladesh.**

| Carcinogenic  Risk |  | **Children** |  |  |  | **Adults** | | | |
| --- | --- | --- | --- | --- | --- | --- | --- | --- | --- |
|  |  | **CR _ingestion_** | **CR _Inhalation_** | **CR _Dermal_** | **TCR** | **CR _ingestion_** | **CR _Inhalation_** | **CR _Dermal_** | **TCR** |
| Pb | Min | 1.68E-07 | 2.68E-17 | 5.22E-13 | 1.8E-07 | 9.9E-08 | 7.58E-17 | 9.45E-13 | 9.97E-08 |
|  | Max | 6.72E-07 | 9.70E-17 | 1.88E-12 | 6.72E-07 | 3.5E-07 | 2.73E-16 | 3.40E-12 | 3.59E-07 |
|  | Mean | 3.50E-07 | 5.04E-17 | 9.80E-13 | 3.50E-07 | 1.87E-07 | 1.42E-16 | 1.77E-12 | 1.87E-07 |
| Cd | Min | 8.53E-08 | 4.12E-18 | 3.96E-09 | 2.2E-07 | 4.56E-08 | 1.16E-17 | 7.16E-09 | 5.28E-08 |
|  | Max | 7.37E-07 | 3.56E-17 | 3.42E-08 | 1.94E-06 | 3.94E-07 | 1.00E-16 | 6.19E-08 | 4.56E-07 |
|  | Mean | 2.53E-07 | 1.22E-17 | 1.17E-08 | 6.6E-07 | 1.35E-07 | 3.45E-17 | 2.12E-08 | 1.56E-07 |
| Cr | Min | 1.85E-05 | 4.52E-16 | 6.75E-16 | 1.85E-05 | 1.64E-06 | 2.12E-16 | 6.23E-08 | 1.71E-06 |
|  | Max | 3.64E-05 | 8.90E-16 | 1.36E-15 | 3.64E-05 | 3.24E-06 | 4.18E-16 | 1.22E-07 | 3.36E-06 |
|  | Mean | 2.58E-05 | 6.31E-16 | 7.09E-16 | 2.58E-05 | 2.29E-06 | 2.96E-16 | 8.69E-08 | 2.38E-06 |
| As | Min | 1.13E-05 | 3.33E-15 | 7.74E-08 | 1.14E-05 | 6.06E-06 | 9.39E-15 | 1.40E-07 | 6.20E-06 |
|  | Max | 4.09E-05 | 1.20E-14 | 2.80E-07 | 4.30E-05 | 2.19E-05 | 3.30E-14 | 5.06E-07 | 2.24E-05 |
|  | Mean | 1.98E-05 | 5.81E-15 | 1.35E-07 | 1.99E-05 | 1.05E-05 | 1.64E-14 | 2.44E-07 | 1.08E-05 |
| Ni | Min | 5.44E-05 | 9.15E-15 | 7.53E-06 | 6.19E-05 | 2.46E-05 | 1.87E-15 | 1.15E-05 | 3.63E-05 |
|  | Max | 1.33E-04 | 2.24E-14 | 1.84E-05 | 1.52E-04 | 7.14E-05 | 5.42E-15 | 3.34E-05 | 1.04E-04 |
|  | Mean | 7.38E-05 | 1.24E-14 | 1.02E-05 | 8.40E-05 | 3.94E-05 | 3.00E-15 | 1.84E-05 | 5.79E-07 |
