# Supplementary material for: Heavy metal footprints in landfill-proximate soils of Jashore, Bangladesh: An index-based risk assessment
Source: PLoS One. 2026 May 21;21(5):e0349757. doi: 10.1371/journal.pone.0349757 (PMC13193546; doi:10.1371/journal.pone.0349757)
Supplement: S10 Table — (DOCX) [file pone.0349757.s010.docx]

**S10 Table. Pearson correlation matrix of heavy metals in the landfill area.**

|  | **As** | **Hg** | **Cd** | **Pb** | **Cr** | **Zn** | **Co** | **Ni** | **Cu** | **Mn** | **Fe** |
| --- | --- | --- | --- | --- | --- | --- | --- | --- | --- | --- | --- |
| **As** | 1 |  |  |  |  |  |  |  |  |  |  |
| **Hg** | 0.105 | 1 |  |  |  |  |  |  |  |  |  |
| **Cd** | 0.036 | 0.610* | 1 |  |  |  |  |  |  |  |  |
| **Pb** | 0.02 | 0.287 | 0.638* | 1 |  |  |  |  |  |  |  |
| **Cr** | 0.714** | 0.274 | 0.4 | 0.395 | 1 |  |  |  |  |  |  |
| **Zn** | -0.232 | 0.342 | 0.418 | 0.695** | 0.182 | 1 |  |  |  |  |  |
| **Co** | 0.411 | -0.215 | 0.064 | -0.098 | 0.563* | -0.464 | 1 |  |  |  |  |
| **Ni** | 0.459 | 0.101 | 0.51 | 0.233 | 0.541* | -0.213 | 0.642** | 1 |  |  |  |
| **Cu** | 0.247 | 0.306 | 0.618* | 0.587* | 0.751** | 0.449 | 0.417 | 0.317 | 1 |  |  |
| **Mn** | 0.788** | 0.021 | -0.031 | -0.066 | 0.509 | -0.163 | 0.352 | 0.487 | 0.115 | 1 |  |
| **Fe** | 0.583* | -0.069 | -0.019 | -0.037 | 0.719** | -0.39 | 0.860** | 0.5 | 0.473 | 0.402 | 1 |

* Correlation is significant at the 0.01 level (2-tailed).

** Correlation is significant at the 0.05 level (2-tailed).
