# Supplementary figures and images for: Heavy metal footprints in landfill-proximate soils of Jashore, Bangladesh: An index-based risk assessment
Source: PLoS One. 2026 May 21;21(5):e0349757. doi: 10.1371/journal.pone.0349757 (PMC13193546; doi:10.1371/journal.pone.0349757)

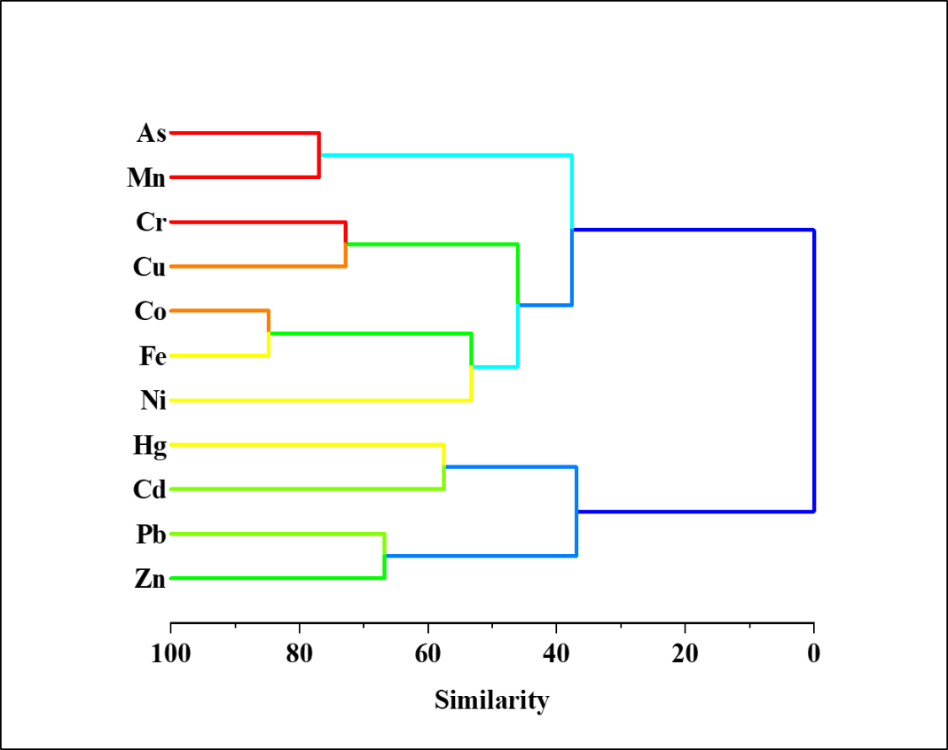


**S1 Fig. Hierarchical clustering analysis of metals in the soil of landfill areas.**

Supplement: S1 Fig — (DOCX) [file pone.0349757.s011.docx]
