# Supplementary material for: Heavy metal footprints in landfill-proximate soils of Jashore, Bangladesh: An index-based risk assessment
Source: PLoS One. 2026 May 21;21(5):e0349757. doi: 10.1371/journal.pone.0349757 (PMC13193546; doi:10.1371/journal.pone.0349757)
Supplement: Attachment — Submitted filename: PONE-D-25-27215R1 Response to reviewers.docx [file pone.0349757.s013.docx]

**Response to Reviewers**

Manuscript ID: PONE-D-25-27215

Title: **Heavy metal footprints in landfill-proximate soils of Jashore, Bangladesh: An index-based risk assessment**

Dear Academic Editor and Reviewers,

We sincerely thank you for your valuable comments and suggestions on our manuscript. We have carefully considered each point raised and revised the manuscript accordingly. Below, we provide detailed responses to the reviewers’ comments. The changes made in the manuscript are highlighted with tracked changes in the revised version.

Comments from PLOS Editorial Office:

1.Please ensure that your manuscript meets PLOS ONE's style requirements, including those for file naming. The PLOS ONE style templates can be found at

<https://journals.plos.org/plosone/s/file?id=wjVg/PLOSOne_formatting_sample_main_body.pdf> and <https://journals.plos.org/plosone/s/file?id=ba62/PLOSOne_formatting_sample_title_authors_affiliations.pdf>

Authors’ response:

We have carefully reviewed and updated the manuscript to ensure full compliance with all **PLOS ONE formatting, style, and file-naming requirements** following the official journal templates. The manuscript, figures, tables, references, and supporting files have been revised accordingly.

2. In your Methods section, please provide additional information regarding the permits you obtained for the work. Please ensure you have included the full name of the authority that approved the field site access and, if no permits were required, a brief statement explaining why.

Authors’ response:

Thank you for this valuable comment. We have added a statement in the Methods section to clarify field access and permit requirements. Soil sampling was conducted in publicly accessible areas surrounding the landfill and on privately owned agricultural land with the voluntary consent of the landowners. No formal permits were required from governmental or institutional authorities, as the study did not involve protected sites or regulated activities. The relevant clarification has now been added to the revised manuscript.

3. Thank you for stating the following financial disclosure:

“Ref No-SRG243321 (2024-2025 FY), Ministry of Science and Technology, The Peoples' Republic of Bangladesh”

Please state what role the funders took in the study.  If the funders had no role, please state: "The funders had no role in study design, data collection and analysis, decision to publish, or preparation of the manuscript."

If this statement is not correct you must amend it as needed.

Please include this amended Role of Funder statement in your cover letter; we will change the online submission form on your behalf.

Authors’ response:

Thanks for the comment. The funders had no role in study design, data collection and analysis, decision to publish, or preparation of the manuscript.

4. We note that your Data Availability Statement is currently as follows: All relevant data are within the manuscript and in Supporting Information files.

Please confirm at this time whether or not your submission contains all raw data required to replicate the results of your study. Authors must share the “minimal data set” for their submission. PLOS defines the minimal data set to consist of the data required to replicate all study findings reported in the article, as well as related metadata and methods (<https://journals.plos.org/plosone/s/data-availability#loc-minimal-data-set-definition>).

For example, authors should submit the following data:

- The values behind the means, standard deviations and other measures reported;

- The values used to build graphs;

- The points extracted from images for analysis.

Authors do not need to submit their entire data set if only a portion of the data was used in the reported study.

If your submission does not contain these data, please either upload them as Supporting Information files or deposit them to a stable, public repository and provide us with the relevant URLs, DOIs, or accession numbers. For a list of recommended repositories, please see <https://journals.plos.org/plosone/s/recommended-repositories>.

If there are ethical or legal restrictions on sharing a de-identified data set, please explain them in detail (e.g., data contain potentially sensitive information, data are owned by a third-party organization, etc.) and who has imposed them (e.g., an ethics committee). Please also provide contact information for a data access committee, ethics committee, or other institutional body to which data requests may be sent. If data are owned by a third party, please indicate how others may request data access.

Authors’ response:

Thanks for the suggestion. All relevant values are mentioned within the manuscript and supporting information file.

5. We note that Figures 1,2 and 4 in your submission contain map/satellite images which may be copyrighted. All PLOS content is published under the Creative Commons Attribution License (CC BY 4.0), which means that the manuscript, images, and Supporting Information files will be freely available online, and any third party is permitted to access, download, copy, distribute, and use these materials in any way, even commercially, with proper attribution. For these reasons, we cannot publish previously copyrighted maps or satellite images created using proprietary data, such as Google software (Google Maps, Street View, and Earth). For more information, see our copyright guidelines: <http://journals.plos.org/plosone/s/licenses-and-copyright>.

 We require you to either (1) present written permission from the copyright holder to publish these figures specifically under the CC BY 4.0 license, or (2) remove the figures from your submission:

 1. You may seek permission from the original copyright holder of Figures 1,2 and 4 to publish the content specifically under the CC BY 4.0 license.

 We recommend that you contact the original copyright holder with the Content Permission Form (<http://journals.plos.org/plosone/s/file?id=7c09/content-permission-form.pdf>) and the following text:

“I request permission for the open-access journal PLOS ONE to publish XXX under the Creative Commons Attribution License (CCAL) CC BY 4.0 (<http://creativecommons.org/licenses/by/4.0/>). Please be aware that this license allows unrestricted use and distribution, even commercially, by third parties. Please reply and provide explicit written permission to publish XXX under a CC BY license and complete the attached form.”

 Please upload the completed Content Permission Form or other proof of granted permissions as an "Other" file with your submission.

 In the figure caption of the copyrighted figure, please include the following text: “Reprinted from [ref] under a CC BY license, with permission from [name of publisher], original copyright [original copyright year].”

 2. If you are unable to obtain permission from the original copyright holder to publish these figures under the CC BY 4.0 license or if the copyright holder’s requirements are incompatible with the CC BY 4.0 license, please either i) remove the figure or ii) supply a replacement figure that complies with the CC BY 4.0 license. Please check copyright information on all replacement figures and update the figure caption with source information. If applicable, please specify in the figure caption text when a figure is similar but not identical to the original image and is therefore for illustrative purposes only.

The following resources for replacing copyrighted map figures may be helpful:

 USGS National Map Viewer (public domain): <http://viewer.nationalmap.gov/viewer/>

The Gateway to Astronaut Photography of Earth (public domain): <http://eol.jsc.nasa.gov/sseop/clickmap/>

Maps at the CIA (public domain): <https://www.cia.gov/library/publications/the-world-factbook/index.html> and <https://www.cia.gov/library/publications/cia-maps-publications/index.html>

NASA Earth Observatory (public domain): <http://earthobservatory.nasa.gov/>

Landsat: <http://landsat.visibleearth.nasa.gov/>

USGS EROS (Earth Resources Observatory and Science (EROS) Center) (public domain): [http://eros.usgs.gov/#](http://eros.usgs.gov/)

Natural Earth (public domain): [http://www.naturalearthdata.com/](http://track.editorialmanager.com/CL0/http:%2F%2Fwww.naturalearthdata.com%2F/1/010f019ada83187e-22fc51b0-7766-4764-b5d7-a7a73da4ad5a-000000/BwMRyB4zOV-UdYTGEA2Wk0KVTqts8cA2qgkz2C3fUk4=239)

Authors’ response:

Thank you for the comment. We confirm that Figure 1,2,4 was created by the authors using ArcGIS software (ArcGIS Desktop 10.8, including ArcMap). These datasets are openly licensed and compatible with the CC BY 4.0 license. No copyright or proprietary sources were used.

6. Please ensure that you refer to Figure 5 in your text as, if accepted, production will need this reference to link the reader to the figure.

Authors’ response:

Thanks for the comment. Fig 5 has been cited in the revised manuscript.

7. If the reviewer comments include a recommendation to cite specific previously published works, please review and evaluate these publications to determine whether they are relevant and should be cited. There is no requirement to cite these works unless the editor has indicated otherwise.

Authors’ response:

Thank you for this guidance. In the present review process, the reviewer did not recommend citing any specific previously published works. Therefore, no additional citations were added on this basis. However, we carefully reviewed the manuscript to ensure that all relevant and appropriate literature has already been adequately cited.

Additional Editor Comments:

Please provide justification - how 15 samples are sufficient to calculate environmental and human health risk index? How many sub-samples were collected to make 15 composite samples? What is the homogeneity of soil properties within sub-sample collection points? This greatly influences your risk indices.

Authors’ response:

A total of **15 composite soil samples (3 sub-samples)** were collected using a **stratified land-use–based sampling approach** encompassing agricultural land, school areas, residential zones, and woodland surrounding the municipal treatment plant. These land-use categories represent distinct ecological and human exposure settings relevant to environmental and human health risk assessment. The environmental contamination indices and human health risk indices applied in this study (e.g., CF, I_geo_, EF, HQ, HI, CR) rely on average contaminant concentrations rather than inferential statistical testing. Consequently, the reliability of these indices depends primarily on the **representativeness of sampling locations and the reduction of small-scale variability**, rather than on large sample numbers. Composite sampling is therefore widely used and accepted in soil risk-assessment studies.

Each composite sample was prepared from **3 sub-samples (triplicate)** collected at each site from the surface soil layer (0-20 cm). The sub-samples were thoroughly mixed to obtain one homogeneous composite sample, which reduces localized heterogeneity and analytical uncertainty. To ensure homogeneity within each sub-sampling point, samples were collected from areas with **uniform land use, similar topography, consistent soil depth, comparable soil texture (clay loam) and color, and no visible recent disturbance**. All sub-sampling locations within a site shared similar environmental and management conditions, supporting the assumption of intra-site soil homogeneity.

Reviewers' comments:

Reviewer's Responses to Questions

**Comments to the Author**

1. Is the manuscript technically sound, and do the data support the conclusions?

The manuscript must describe a technically sound piece of scientific research with data that supports the conclusions. Experiments must have been conducted rigorously, with appropriate controls, replication, and sample sizes. The conclusions must be drawn appropriately based on the data presented.

Reviewer #1: Yes

2. Has the statistical analysis been performed appropriately and rigorously?

Reviewer #1: N/A

3. Have the authors made all data underlying the findings in their manuscript fully available?

The [PLOS Data policy](http://track.editorialmanager.com/CL0/http:%2F%2Fwww.plosone.org%2Fstatic%2Fpolicies.action%23sharing/1/010f019ada83187e-22fc51b0-7766-4764-b5d7-a7a73da4ad5a-000000/09uix0mV2pQ-qeUl-GYk9IgLu5_n6J-PYBuSfEiXeV8=239) requires authors to make all data underlying the findings described in their manuscript fully available without restriction, with rare exception (please refer to the Data Availability Statement in the manuscript PDF file). The data should be provided as part of the manuscript or its supporting information, or deposited to a public repository. For example, in addition to summary statistics, the data points behind means, medians and variance measures should be available. If there are restrictions on publicly sharing data—e.g. participant privacy or use of data from a third party—those must be specified.

Reviewer #1: No

4. Is the manuscript presented in an intelligible fashion and written in standard English?

PLOS ONE does not copyedit accepted manuscripts, so the language in submitted articles must be clear, correct, and unambiguous. Any typographical or grammatical errors should be corrected at revision, so please note any specific errors here.

Reviewer #1: Yes

5. Review Comments to the Author

Please use the space provided to explain your answers to the questions above. You may also include additional comments for the author, including concerns about dual publication, research ethics, or publication ethics. (Please upload your review as an attachment if it exceeds 20,000 characters)

Reviewer #1:

Heavy metal footprints in landfill-proximate soils of Jashore, Bangladesh: An index-based risk assessment

Sanjida Sultana Santa 1, Md Kamal Hossain 2,3*, Kowshik Das Karmaker 4, Mohammad Moniruzzaman2,3 , Md. Harunor Rashid Khan 1
Bernardo Sepúlveda.

My observations are:

1. There are several situations in which the plural is used in sentences and should be singular, a couple of examples are: “The average concentrations of Hg, Cd, Pb, Zn, and Cu were higher than, if we are talking about the concept of concentration it should say CONCENTRATION, if we are talking about more than one concentration for each element it would admit the plural; but, it does not seem to be the case; otherwise it should say how many concentrations of each element it is describing. Another example: “While the current contamination levels LEVEL suggest a…”… the “current pollution (I like this word) LEVEL …., etc.

**Author’s response:**

We thank the reviewer for pointing out the grammatical ambiguity. The text has been revised to clarify singular and plural usage. Specifically, “average concentrations” has been revised to “average concentration of each metal” to indicate one mean value per element, and “contamination levels” have been corrected to “contamination level” where the overall pollution status is discussed.

2. In the conclusion it says… “…certain sampling points…”. If these points produce a conclusion, these should be mentioned in some way and in the discussion it should be said what they are or why it is important to be at those points.

**Author’s response:**

We thank the reviewer for this helpful comment. In the revised manuscript, the conclusion has been clarified to indicate that elevated concentrations above shale values were observed **at sampling locations closer to the landfill.** The wording in the conclusion has been revised accordingly to improve clarity and traceability.

3. Regarding the conclusion, it has elements that are not useful for this purpose; I suggest you objectify it to the specific achievements of the work, eliminating phrases that summarize the methodology and discussion. Projections are fine. I propose something like this, it is just an example:

“The average concentration of Hg, Cd, Pb, Zn and Cu was higher than the corresponding values in shale; the concentration of other metals and metalloids was lower than those un shale, although higher than shale level in the …. ¿points?. This results suggest that metal pollution in solils is inked with the regular operation of the landfill. The current level of the contamination ranges from no-polluted to moderately polluted. It is evident that if waste combustión at the landfill continues at the present rate, contamination level are likely to increase in the near future. There was an imbalance in the concentration of metals in the soil samples, varying according to the distance from the landfill and the topography of the terrain. Further research is needed, considering the impact of climatic conditions, human activity and the expansion of the sampling área. It is hypothesised that the burning of waste in landfill is the main factor contributing to the presence of trace elements. This information may prove valuable to researchers and environmental activists studying ecological áreas affected by open dumping of municipal waste, both locally and globally.”

**Author’s response:**

We thank the reviewer for summarizing the main findings of the study. The conclusion has been revised to improve clarity, grammatical consistency, and scientific precision, and to explicitly link metal pollution in soils to landfill operation, waste combustion, distance from the landfill, and topographic effects. Redundant wording has been streamlined, singular–plural inconsistencies corrected, and vague expressions clarified.

4. In 2.5, there is mention of the software used for the analysis; however, I didn't find the names of the analyses, for example, "Student's t," analysis of variance, Fisher's test, etc. If that's not the case, I haven't mentioned anything; perhaps I did´t see it.

**Author’s response:**

Thank you for your insightful comment. We have revised Section 2.5 (Statistical Analysis) to explicitly include the names of the statistical tests applied, including the one-sample Student’s *t*-test used to compare metal concentrations with shale background values. We have also specified the software used for these analyses (IBM SPSS Statistics 26). This addition clarifies the statistical methods employed and enhances the transparency of the data analysis.

5. I have a reasonable doubt. The results state that certain concentrations are higher or lower than others; however, there are no statistical indicators; they are merely statements based on judgment. I must say this because statistical tests were supposedly applied; therefore, some indicator, such as "p" should have been calculated to confirm the differences between the data.

**Author’s response:**

Thank you for your insightful comment. In response, we have now performed one-sample Student’s *t*-tests comparing the mean concentrations of heavy metals to shale background values. The results, including *p*-values, have been incorporated into the revised manuscript to objectively determine which metals show statistically significant enrichment or depletion.

Specifically, we added the following statement to the Results section: “One-sample Student’s *t*-tests were conducted to compare the mean concentrations of heavy metals with shale background values. The results indicated that Cd, Pb, Zn, and Cu concentrations were significantly higher than the shale values (*p* < 0.05), suggesting anthropogenic enrichment. In contrast, Cr, Co, Ni, Mn, and Fe showed significantly lower concentrations than the corresponding shale values (*p* < 0.05). No statistically significant difference was observed for As and Hg (*p* > 0.05).”

This addition provides the necessary statistical evidence to support our conclusions and strengthens the validity of our findings.

6. In Table 1... didn't you analysed triplicate samples for each element? I mention this because elsewhere in the text, the value for an element is referred to as "an average." Therefore, if it is an average, it is because there is a statistical error or SD. In these cases the correct nomenclature is xx+xx format. For example, it has been mentioned that 49.6 (unit) is an average of Cr, but the correct value should be 46.9+??.

Authors’ response:

Thank you for this important observation. We confirm that all samples were analyzed in triplicate for each element to ensure analytical precision and quality control. In the manuscript, the results were presented only as mean values, which may have caused ambiguity. Following the reviewer’s suggestion, we have now revised the corresponding text to report the data in the appropriate mean ± standard deviation (SD) format (e.g., 46.9 ± 10.06 mg/kg) wherever averages are mentioned. The manuscript has been updated accordingly.

7. The structure of the tables could be improved, using single-spaced lines and closer columns, specifically in Table 1. Table 3 could be improved, as it repeats the word COEFFICIENT three times; It could be mentioned as a heading on the top line, and the columns could be closer together. All of this would optimize the space occupied.

Coefficient (unit)
A B C
xxxx xxxx xxxx

**Author’s response:**

We thank the reviewer for the valuable suggestions regarding the table formatting. We have revised Table 1 to use single-spaced lines and adjusted the column spacing to make the layout more compact and reader-friendly. Similarly, Table 3 has been reformatted by consolidating the repeated word “COEFFICIENT” into a single heading at the top of the columns, and the columns have been brought closer together to optimize space without compromising readability.
