# Supplementary material for: Heavy metal footprints in landfill-proximate soils of Jashore, Bangladesh: An index-based risk assessment
Source: PLoS One. 2026 May 21;21(5):e0349757. doi: 10.1371/journal.pone.0349757 (PMC13193546; doi:10.1371/journal.pone.0349757)
Supplement: Attachment — Submitted filename: PONE-D-25-27215 R2 Response to reviwers.docx [file pone.0349757.s014.docx]

**Response to Reviewers**

Manuscript ID: PONE-D-25-27215R1

Title: **Heavy metal footprints in landfill-proximate soils of Jashore, Bangladesh: An index-based risk assessment**

Additional Editor Comments:

Dear Authors,

Thank you for your patience, we have received major revision from additional reviewer as your original reviewers were not available to track your alterations. Hope to receive your revised version.

**Response to editor:** Thank you for your valuable time and for arranging the additional review. We have carefully revised the manuscript in accordance with the reviewer’s comments and have included a detailed response. We have highlighted the changes in red color which we made based on their feedback in the marked manuscript.

[Note: HTML markup is below. Please do not edit.]

Reviewer's Responses to Questions

Comments to the Author

1. If the authors have adequately addressed your comments raised in a previous round of review and you feel that this manuscript is now acceptable for publication, you may indicate that here to bypass the “Comments to the Author” section, enter your conflict of interest statement in the “Confidential to Editor” section, and submit your "Accept" recommendation.

Reviewer #2: (No Response)

Reviewer #3: All comments have been addressed

Reviewer #4: (No Response)

2. Is the manuscript technically sound, and do the data support the conclusions?

The manuscript must describe a technically sound piece of scientific research with data that supports the conclusions. Experiments must have been conducted rigorously, with appropriate controls, replication, and sample sizes. The conclusions must be drawn appropriately based on the data presented.

Reviewer #2: Yes

Reviewer #3: Yes

Reviewer #4: Partly

3. Has the statistical analysis been performed appropriately and rigorously?

Reviewer #2: No

Reviewer #3: Yes

Reviewer #4: (No Response)

4. Have the authors made all data underlying the findings in their manuscript fully available?

The PLOS Data policy requires authors to make all data underlying the findings described in their manuscript fully available without restriction, with rare exception (please refer to the Data Availability Statement in the manuscript PDF file). The data should be provided as part of the manuscript or its supporting information, or deposited to a public repository. For example, in addition to summary statistics, the data points behind means, medians and variance measures should be available. If there are restrictions on publicly sharing data—e.g. participant privacy or use of data from a third party—those must be specified.

Reviewer #2: No

Reviewer #3: Yes

Reviewer #4: (No Response)

5. Is the manuscript presented in an intelligible fashion and written in standard English?

PLOS ONE does not copyedit accepted manuscripts, so the language in submitted articles must be clear, correct, and unambiguous. Any typographical or grammatical errors should be corrected at revision, so please note any specific errors here.

Reviewer #2: Yes

Reviewer #3: No

Reviewer #4: Yes

6. Review Comments to the Author

Please use the space provided to explain your answers to the questions above. You may also include additional comments for the author, including concerns about dual publication, research ethics, or publication ethics. (Please upload your review as an attachment if it exceeds 20,000 characters)

**Point-by-point response to reviewer**

Reviewer #2: Please address the following comments in your second revision:

1. While you have justified the use of 15 composite samples based on stratified land use, an open dumping site processing 50 tonnes of waste daily likely creates highly heterogeneous contamination plumes. Please provide a more rigorous technical justification in the "Soil Sampling" section explaining why 15 points are sufficient to capture the full extent of this variability without missing localized "hotspots."

**Response to reviewer:** Thank you for this important and technically insightful comment. We agree that landfill environments can generate highly heterogeneous contamination patterns due to variable waste composition and leachate dispersion. To address this concern, we have strengthened the justification of our sampling design in the revised manuscript (Soil Sampling section). Specifically, a stratified sampling approach was adopted based on land-use categories and directional gradients surrounding the landfill, ensuring coverage of key exposure zones (agricultural, residential, school, and wild land). Each of the 15 sampling points represents a composite of three sub-samples, which reduces microscale variability and improves representativeness. The adequacy of the sampling framework is supported by the moderate to high coefficients of variation (e.g., Cd: 80.3%, Zn: 77.1%), indicating that spatial heterogeneity was effectively captured. Importantly, consistent identification of high-contamination zones (e.g., Site ID-5 and ID-9) across multiple independent indices (CF, PLI, RI) suggests that major contamination hotspots were successfully detected.

2. Your study is restricted to surface soil (0–20 cm). Given the high organic load and leachate production mentioned in your introduction, please discuss the potential for vertical migration of metals into deeper soil profiles, particularly in the tropical, high-rainfall environment of Jashore.

**Response to reviewer:** Thank you for this valuable comment. We agree that vertical migration of heavy metals is an important consideration, particularly in landfill-affected environments. We have incorporated a detailed discussion in the “Distribution of heavy metals” to address the potential for downward migration of metals. Specifically, we explain that in a tropical monsoon setting such as the study area, characterized by high annual rainfall, landfill-generated leachate can infiltrate the soil profile and facilitate the vertical transport of metals. Additionally, we have acknowledged this as a limitation of the current study (which focuses on surface soils, 0–20 cm) and emphasized the need for future investigations incorporating subsurface soil profiling to better understand vertical distribution and long-term transport mechanisms.

3. Interpolation Accuracy: In Figures 2 and 4, you present spatial distribution maps for metals and risk indices. You must specify the mathematical interpolation method used (e.g., Kriging, IDW) in Section 2.5 to ensure these maps are not over-extrapolating from a limited dataset.

**Response to reviewer:** Thank you for this suggestion. We have now explicitly stated in Statistical analysis section that Inverse Distance Weighting (IDW) interpolation was applied in ArcMap 10.8 to generate spatial distribution maps (Figures 2 and 4). The choice of IDW was based on its suitability for relatively small datasets and its ability to preserve local variation without excessive smoothing.

4. Local Control Sites: Currently, your primary reference for contamination is global average "shale values". It is expected that authors use a local "control" site (soil with similar geological properties but distant from landfill influence). Please explain why a local reference was not established or provide data if available.

**Response to reviewer:** We appreciate this important point. Due to logistical constraints and the widespread influence of anthropogenic activities in the surrounding region, identifying a truly unaffected local control site with comparable soil properties was challenging. The use of global shale values [28] enables cross-study comparability and is widely employed in the Bangladeshi literature [19, 61]. The study area and its surroundings are influenced by multiple sources, including landfill activities, agriculture, and nearby industrial operations, making it challenging to identify an uncontaminated reference location. While the use of shale values is a widely accepted approach in similar studies, it may introduce some uncertainty. Therefore, future research should incorporate site-specific background values from geologically similar but minimally impacted areas to improve accuracy.

5. Please include a table in the Supporting Information listing the Limit of Detection (LOD) and Limit of Quantification (LOQ) for each of the 11 metals analyzed via ICP-MS and AAS. This is essential to verify the precision of your minimum reported values.

**Response to reviewer:** Thanks for the comment. The Limit of Detection (LOD) and Limit of Quantification (LOQ) level for each of the 11 metals analyzed has been added in the supplementary S2a table.

6. You report that the Total Cancer Risk (TCR) for Nickel (Ni) in children exceeds permissible thresholds. Given that the school field was leveled using riverside sediment, please expand your discussion on the specific local pathways for this group. Are these children in direct contact with the sediment during daily activities?

**Response to reviewer:** Thank you for this valuable comment. We have expanded the discussion in carcinogenic risk to clarify site-specific exposure pathways. The school field was leveled using contaminated river sediment, which is frequently used by children for daily outdoor activities such as playing and sports. Thus, direct ingestion of soil/dust, dermal contact, and incidental inhalation of resuspended particles are likely dominant exposure pathways for children. These behaviors significantly increase exposure frequency and explain the elevated TCR for Ni observed in children compared to adults.

7.Your health risk models (HQ, HI, TCR) generally assume 100% bioavailability. Please include a brief discussion on how the bioavailable fraction of these metals might differ from the total concentrations measured, as this is a key factor in screening-level evaluations.

**Response to reviewer:** We thank the reviewer for this insightful comment. We have added a statement in the Health Risk Assessment section acknowledging that HQ, HI, and TCR calculations assume 100% bioavailability, and that actual risks may be lower depending on the bio-accessible fraction of metals.

8. In Section 3.6, you attribute metals in PC2 (Hg, Cd, Pb, Cr) to anthropogenic origins such as waste management. Please explicitly reference the specific correlation coefficients from S10 Table in the text to reinforce the statistical link between these elements and waste burning.

**Response to reviewer:** Thanks for the comment. This correlation has been added in the PCA analysis section.

I look forward to receiving your point-by-point response and the revised manuscript.

Reviewer #3: After having a close look I understand that the manuscript has been properly addressed following the comments. No other comments from myside.

**Response to reviewer:** Thank you very much for your careful evaluation of the manuscript and for acknowledging that the revisions have adequately addressed the previous comments. We sincerely appreciate your time and constructive feedback, which has helped improve the quality of our work.

Reviewer #4: Heavy metals that are defined as toxic to the site, As, Hg, Cd, Pb, Zn, Cu and Mn needs to be compared with either standard background values of the area or with other available standard values. According to the FAO/WHO standard value, only Cu value seems to be in the toxic area, exceeding the normal limit. Therefore, I could not find any other convincing fact to explain the available values of above-mentioned elements as in danger zone.

**Response to reviewer:** Thank you for your valuable and constructive comment. We fully agree that the comparison of heavy metal concentrations with appropriate background or standard values is essential for accurately interpreting contamination levels. However, in the present study, establishing site-specific background values was challenging due to the widespread influence of anthropogenic activities (e.g., landfill operations, agriculture, and nearby industrial sources) across the study area and its surroundings. These factors limited our ability to identify a truly uncontaminated reference site with comparable geochemical characteristics. Furthermore, there is currently no established national soil quality standard for Bangladesh, and available FAO/WHO guideline values do not comprehensively cover all the analyzed metals in this study. Therefore, we adopted global shale values as background references, which is a widely accepted approach in similar environmental assessments and has been applied in previous studies conducted in Bangladesh (Refs. [19, 61]). We acknowledge that the use of generalized shale values may introduce some level of uncertainty. In light of this, a statement has been included in the revised manuscript highlighting this limitation. Future studies are recommended to incorporate site-specific baseline values from geologically similar but minimally impacted areas to enhance the accuracy and robustness of contamination assessment.

7. PLOS authors have the option to publish the peer review history of their article (what does this mean?). If published, this will include your full peer review and any attached files.

If you choose “no”, your identity will remain anonymous but your review may still be made public.

Do you want your identity to be public for this peer review? For information about this choice, including consent withdrawal, please see our Privacy Policy.

Reviewer #2: Yes: Gandhiv Kafle

Reviewer #3: Yes: Peiman Zandi

Reviewer #4: No
